# RefAHL: a curated quorum sensing reference linking diverse LuxI-type signal synthases with their acyl-homoserine lactone products

**DOI:** 10.1128/mra.00407-25

**Published:** 2025-05-22

**Authors:** Amy L. Schaefer, Ethan G. Murdock, Dale A. Pelletier, Caroline S. Harwood, E. Peter Greenberg, Aaron W. Puri

**Affiliations:** 1 Department of Microbiology, University of Washington 312771, Seattle, Washington, USA; 2 Department of Chemistry, Henry Eyring Center for Cell and Genome Science, University of Utah 7060, Salt Lake City, Utah, USA; 3 Biosciences Division, Oak Ridge National Laboratory 6146, Oak Ridge, Tennessee, USA; Indiana University Bloomington, Bloomington, Indiana, USA

**Keywords:** quorum sensing, sociomicrobiology, AHL, acyl-homoserine lactone

## Abstract

Some bacteria use acyl-homoserine lactone (AHL) signals in quorum sensing, a type of cell-cell communication. Here, we present “RefAHL,” an updated, curated collection of LuxI-type AHL synthases with their AHL products and associated metadata. RefAHL is publicly available as a community resource to help catalog LuxI-type diversity encoded in (meta) genomic data.

## ANNOUNCEMENT

Many bacteria use acyl-homoserine lactone (AHL) signal compounds, produced by the LuxI-family of synthases and detected by the LuxR-family of transcriptional regulators, in cell density-dependent gene regulation called quorum sensing (QS) (1–3). QS system specificity is conferred by the AHL acyl group moiety, and about 35 different AHLs have been described, varying in acyl side chain length (4–20 carbons) and substitution. In pair-wise comparisons, there is low (20%–25%) amino acid sequence identity between most LuxI homologs, even between those that synthesize the same AHL signal (3). Metagenomic sequence analysis suggests a large diversity of LuxI homologs, with over >6,000 unique *luxR-luxI* pairs identified (4). However, for the vast majority of these homologs, the AHL signal product is undefined.

**Fig 1:**
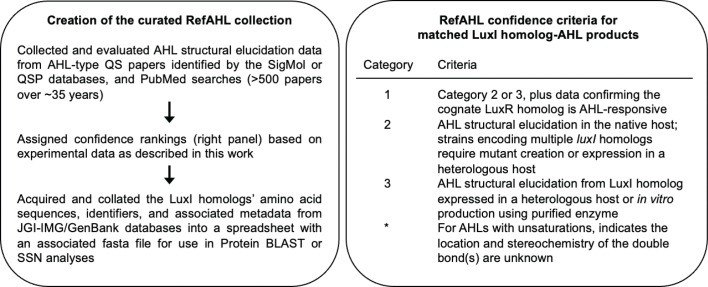
(Left) Summary of the development of the RefAHL quorum sensing reference. (Right) Description of the RefAHL confidence category criteria.

Since the discovery of the first AHL-type QS system, *Vibrio fischeri* LuxI and its AHL 3-oxo-hexanoyl homoserine lactone (5–7), there has been considerable effort in the field to identify additional LuxI homologs and their AHL products from other bacteria. Using the SigMol (8) and the QS-related protein (QSP) (9) databases as starting points, we updated and curated a list of 134 LuxI homologs experimentally shown to synthesize an AHL of well-supported structure and refer to this collection as “RefAHL” (Fig. 1 and associated data). Our definition of well-supported structure requires specific analytical characterization(s), including mass spectrometry, NMR spectroscopy, isotopically labeled methionine-feeding experiments (10, 11), and/or cognate LuxR homolog-specific activation. Support for AHL structures was assigned a confidence category, depending upon the experimental analyses used (Fig. 1 and associated data). Readers should know that AHL assignments based solely on relaxed specificity bioassays (e.g., *Agrobacterium* TraR-based [12, 13] or *Chromobacterium* CviR-based [14, 15] bioreporters) are not included in RefAHL. Why not? Because these assays can greatly overestimate the relative abundance of minor AHLs known to be synthesized by LuxI-type enzymes (16–18), often leading to misidentification of the relevant AHL signal (discussed in references 10, 19). We also omitted LuxI homologs for which we could not identify the primary AHL product from the published data. We acknowledge that this removes many reported LuxI homologs from inclusion in RefAHL, but we choose to have a smaller, albeit more robust, data set.

RefAHL includes taxonomic (clone library) information, LuxI-homolog amino acid sequences, Integrated Microbial Genomes/GenBank identifiers (20, 21), literature references (with PMID number), the major LuxI signal produced (with PubChem number and isomeric SMILES structure), as well as our confidence rating assignments for the AHL structure associated with each LuxI homolog (Fig. 1). We assume that homoserine lactone stereochemistry is L, and any 3-hydroxy acyl group is R, although this has only been experimentally determined for a handful of AHL signals (22–24).

The RefAHL collection is publicly available in tabular form containing the LuxI homolog protein sequences with detailed metadata, as well as in a machine-readable FASTA format, both of which are hosted on Dryad (https://doi.org/10.5061/dryad.866t1g21s). Researchers can use these data as comparator references in their in-house analysis pipelines or publicly available tools such as protein BLAST (20) or sequence similarity network (SSN) analyses (25) when investigating genomic sequencing data. As an example, we used LuxI comparator sequences present in RefAHL to ascertain that the AHL signal made by the most prevalent LuxI homolog in *Methylobacterium*/*Methylorubrum* genomes was (at the time) undefined—we then focused our efforts to elucidate this novel AHL signal (24). We encourage researchers to contact us with new RefAHL entries to increase the value of this resource over time.

## Data Availability

RefAHL is hosted on Dryad (https://doi.org/10.5061/dryad.866t1g21s) and contains the following files (note that filename date suffixes will change with updated future versions): RefAHL_complete_revYYYYMMDD.xlsx: RefAHL curated list of LuxI homologs with well-supported AHL structures (Tab 1), AHL structural information (Tab 2), and RefAHL confidence category criteria (Tab 3). RefAHL_revYYYYMMDD.fasta: FASTA file containing amino acid sequences of the RefAHL LuxI homologs. We will continue to update and maintain RefAHL for at least 10 years after publication.
