# Erratum for Schaefer et al., “RefAHL: a curated quorum sensing reference linking diverse LuxI-type signal synthases with their acyl-homoserine lactone products”

**DOI:** 10.1128/mra.00644-25

**Published:** 2025-07-14

**Authors:** Amy L. Schaefer, Ethan G. Murdock, Dale A. Pelletier, Caroline S. Harwood, E. Peter Greenberg, Aaron W. Puri

## ERRATUM

Volume 14, no. 6, e00407-25, 2025, https://doi.org/10.1128/mra.00407-25.

Page 2, the following statement should be added to the Acknowledgments section: “This research was supported by NIH grants R35 GM136218 (to E.P.G.) and GM118762 (to A.W.P.), NSF CAREER Award 2339190 (to A.W.P.) and the Genomic Science Program, US Department of Energy, Office of Science, Biological and Environmental Research (to E.P.G., C.S.H., A.L.S., D.A.P.), as part of the Plant Microbe Interfaces Scientific Focus Area (pmi.ornl.gov). Oak Ridge National Laboratory is managed by UT-Battelle LLC for the U.S. Department of Energy under Contract DE-AC05-00OR22725.”

